# The longitudinal effects of faith and meaning on quality of life in US women with breast cancer undergoing chemotherapy

**DOI:** 10.1017/S1478951526102806

**Published:** 2026-06-10

**Authors:** Elizabeth Catherine Conti, Chelsea Gilts Ratcliff, Alejandro Chaoul, Lorenzo Cohen

**Affiliations:** 1 Department of Palliative, Rehabilitation, and Integrative Medicine, The University of Texas MD Anderson Cancer Center, Houston, TX, USA; 2 Department of Psychology, University of Houston, Houston, TX, USA; 3 The Jung Center’s Mind Body Spirit Institute, Houston, TX, USA

**Keywords:** Breast cancer, faith, spirituality, meaning, quality of life

## Abstract

**Objectives:**

This study examined longitudinal associations between spirituality and quality of life (QoL) in women newly diagnosed with breast cancer and undergoing chemotherapy at a large tertiary cancer center.

**Methods:**

Women (*N* = 114) completed measures of spirituality (3 subscales of the Functional Assessment of Chronic Illness Therapy – Spiritual Well-Being Scale: Meaning, Peace, and Faith) and health-related QoL (36-Item Short Form Health Survey) at study entry and 3- and 15-months later. Bias-corrected bootstrap tests were used to examine whether baseline Faith was indirectly associated with mental and physical QoL 15 months later via Meaning and Peace at 3 months.

**Results:**

Baseline Faith was positively associated with Meaning (*β* = .31, *p* = .001) and Peace (*β* = .38, *p* < .001) at 3 months. Both Meaning [*n* = 94, effect = .07 (95% CI: .002, .17)] and Peace [*n* = 93, effect = .13 (95% CI: .02, .28)] mediated the association between Faith and mental QoL at 15 months. When baseline Meaning was controlled, the indirect effect of baseline Faith on mental QoL remained significant, [*n* = 94, effect = .07 (95% CI: .001, .18)], and increases in Meaning over the first 3 months became an even stronger predictor of later mental QoL (*β* = .36, *p* = .004), suggesting that change in meaning during active treatment is an especially important predictor of mental QoL.

**Significance of results:**

Overall, findings demonstrate that Faith is indirectly associated with long-term QoL through early increases in Meaning and Peace. Because 90% of participants were within 6 weeks of diagnosis, the study provided a unique perspective on spirituality during the early treatment period. Clinically, results highlight the importance of early assessment of spiritual well-being and suggest that meaning-focused interventions may enhance long-term QoL for women undergoing chemotherapy.

For individuals diagnosed with breast cancer, treatment poses substantial challenges to quality of life (QoL), particularly in the first year after diagnosis (Montazeri 2008). In addition to the direct side effects of treatment such as pain and nausea, common difficulties include heightened anxiety and psychological distress, fatigue, insomnia, perceived cognitive decline, and sexual dysfunction (Montazeri 2008; Heidary et al. 2023). Additional challenges may include changes in social roles and identity, worries about children and family, and fear of cancer recurrence (Heidary et al. 2023). Collectively, these factors undermine health-related QoL.

Religion and spirituality are coping resources that may bolster health-related QoL in women with breast cancer (Peteet and Balboni 2013). Yet, findings are mixed regarding the direct positive effects of religion and spirituality on health-related QoL, particularly in relation to physical well-being (Hebert et al. 2009; Counted et al. 2018; Peres et al. 2018). Some studies suggest that the constructs of spirituality, including meaning and peace, play a more direct role in bolstering QoL than religion or faith alone (Whitford and Olver 2012; Bai and Lazenby 2015; Bai et al. 2016; Walker et al. 2017). Conversely, spiritual pain has a clear association with adverse physical and mental health outcomes in individuals with cancer (Hebert et al. 2009; Delgado-Guay et al. 2021; Canada et al. 2023).

The Functional Assessment of Chronic Illness Therapy–Spiritual Well-Being Scale (FACIT-Sp) was developed to measure spirituality in diverse oncology patient populations including both religious and nonreligious people (Peterman et al. 2002). The scale comprises 3 subscales: Meaning – assessing the cognitive dimension of spirituality; Peace – assessing affective dimension of spirituality; and Faith – assessing the impact of one’s spiritual beliefs in the context of illness (Canada et al. 2008; Ahmad et al. 2022). Many studies have examined the relative contribution of Meaning, Peace, and Faith, as assessed by the FACIT-Sp, in accounting for variability in QoL among various cancer populations (e.g., Stefanek et al. 2005; Yanez et al. 2009; Bai and Lazenby 2015). Previous research has generally found the Meaning and Peace subscales to have a stronger association with QoL relative to the Faith subscale, leading some to conclude that faith is less consequential to health-related QoL.

However, other investigators have proposed a more nuanced, theoretically informed association among these constructs (Park 2013; Canada et al. 2016). For example, Park (2005) posits that religious faith can be understood as a system which informs the process of meaning-making when faced with situations that challenge one’s previously held beliefs (e.g., receiving a cancer diagnosis). In other words, “a person’s global framework of beliefs (i.e., faith) plays an important role in ascribing significance (i.e., meaning) to events, such as a cancer diagnosis or long-term symptoms” (Canada et al. 2016). Several researchers have explored this model, proposing that Faith indirectly effects QoL as a result of its association with Meaning and Peace (Edmondson et al. 2008; Park et al. 2008; Canada et al. 2016; Merluzzi et al. 2023). Indeed, their findings suggest that Faith is not directly associated with QoL but promotes QoL indirectly by fostering a sense of Meaning and Peace.

Importantly, most studies examining the indirect effect of faith on QoL via meaning and peace have used cross-sectional data, which limits the ability to examine mediation due to the lack of longitudinal measures over time (Maxwell and Cole 2007; Fairchild and McDaniel 2017). It also introduces a challenge of shared variability, given that the Meaning and Peace subscales of the FACIT-Sp include indicators of mental health, which are also aspects of health-related QoL, thus their associations could be considered “tautological” (Koenig and Carey 2025). Modeling spirituality subscales that are “contaminated” with mental health-related content as mediators is one way to remedy this issue (Koenig and Carey 2025).

Additionally, cross-sectional data precludes the ability to examine the impact of faith on *change* in meaning and/or peace during critical times during the cancer treatment experience (e.g., active treatment), and the association of this change with subsequent health-related QoL. One study of ovarian cancer survivors found that changes in spiritual well-being from pretreatment to one-year later were small but common, with 25–30% experiencing a decline and 35–55% experiencing an increase in each facit of spiritual well-being (Davis et al. 2018). Further, change in spiritual well-being scores was associated with mental health 1-year posttreatment. It is reasonable to posit that faith, likened to one’s worldview, may inform the extent to which a person experiences an increase or decrease in their sense of spiritual meaning or peace, and this change may be particularly predictive of subsequent health-related QoL.

Existing research has also focused on cancer survivors, which limits the ability to examine the effects of spirituality early in the disease and treatment process on later meaning, peace, and health outcomes. Longitudinal and prospective studies including newly diagnosed patients are needed to clarify the complex association between these psychosocial factors and subsequent health-related QoL.

## The present study

The present study examined the extent to which Faith early in breast cancer diagnosis and treatment was associated with mental and physical health-related QoL assessed 15 months later by exploring the direct and mediating roles of Meaning and Peace assessed 3-months after study entry. Additional analyses also examined *change* in Meaning and Peace as mediators of the association between Faith and subsequent health-related QoL.

The current study is a secondary analysis of a randomized controlled trial assessing the effects of a Tibetan Yoga program compared to stretching or waitlist control conditions on sleep and fatigue over a 15-month follow-up period (Chaoul et al. 2018). We hypothesized that Faith at study entry (baseline) would be positively associated with Meaning and Peace assessed 3 months later, which in turn would be positively associated with mental and physical health-related QoL at 15 months. Additionally, we hypothesized that higher Faith at study entry would be associated with a greater *increase* in 3-months Meaning and Peace (i.e., controlling for Meaning and Peace at study entry), and greater increase in Meaning and Peace would be associated with higher 15-month mental and physical health-related QoL.

## Methods

### Participants

The sample for the present study is comprised of 114 participants enrolled from Chaoul et al. (2018) who completed the study measures at the time points used in the present study (i.e., FACT-Sp at baseline and 3-months; SF-36 at baseline and 15-months). Recruitment procedures and detailed methodology are outlined in the original study (Chaoul et al. 2018). Eligible participants were English-speaking women over the age of 18 diagnosed with stage I–III breast cancer. They were either currently receiving chemotherapy, scheduled for neoadjuvant or adjuvant chemotherapy, or had completed chemotherapy within the past year. Individuals were excluded if they had lymphedema, deep vein thrombosis, a diagnosed thought disorder (such as schizophrenia), a Mini-Mental State Examination score of 23 or lower, severe mobility limitations, were participating in psychological counseling or support groups, or had engaged in regular yoga practice in the year prior to their breast cancer diagnosis.

### Measures

#### Meaning, peace, and faith (spiritual well-being)

Spiritual well-being was measured with the 3-factor model of the FACIT-Sp (Peterman et al. 2002; Canada et al. 2008). The 3 subscales of Meaning, Peace, and Faith each include 4 items with Likert scale responses from 0 (not at all) to 4 (very much). Examples of items representing each subscale are “I have a reason for living” (Meaning), “I feel a sense of harmony within myself” (Peace), and “I find strength in my faith or spiritual beliefs” (Faith). The subscales demonstrated adequate internal consistency in the present sample (Cronbach α for Meaning > 0.73, Peace > 0.83, Faith > 0.85 at baseline at 3-months).

#### Health-related quality of life

Health-related QoL was measured using the 36-item Short Form Health Survey (SF-36) developed by the Medical Outcomes Study (Ware 1999). This tool captures patients’ self-reported perceptions of their health-related QoL. Responses were collected using both 3-point and 6-point Likert-type scales. The SF-36 includes 8 subscales: physical functioning, role limitations due to physical health, bodily pain, general health, vitality, social functioning, role limitations due to emotional problems, and mental health. For the current study, emphasis was placed on the 2 summary scores – the Physical Component Summary (PCS) and the Mental Component Summary (MCS) – which reflect overall physical and mental health, respectively. Higher scores on the PCS and MCS indicate better perceived health-related QoL. Due to the varied weighting of items across the scales, internal consistency (Cronbach’s *α*) was not calculated for this sample. However, previous studies involving similar populations have demonstrated acceptable reliability for these component scores (Reulen et al. 2006).

### Procedure

Approval for the study protocol was granted by the University of Texas MD Anderson Cancer Center Institutional Review Board (protocol number 2005-0035), and participants were recruited between 2007 and 2012 during routine clinic visits. After providing informed consent, participants completed a baseline assessment and were randomly assigned to 1 of 3 groups: a yoga program, a stretching program, or a waitlist control group. Data collection took place at study entry (baseline), 3 months (immediately following the intervention), 6 months (3 months post-intervention), 9 months (6 months post-intervention), and 15 months (12 months post-intervention). Additional procedural details and a CONSORT diagram outlining participant flow from recruitment through the final follow-up are available in the original study report (Chaoul et al. 2018).

### Statistical methods

Bivariate associations between demographic factors and the primary outcome variables (SF-36 MCS and PCS) were examined to determine relevant covariates. Pearson correlations were conducted for continuous variables, t-tests were conducted for dichotomous categorical variables, and ANOVA was conducted for multi-categorical variables. Demographic variables associated with the outcome (*p* < .05), the baseline level of the outcome variable, and treatment group (yoga, stretching, or waitlist) were included in the initial models; covariates that did not account for significant variance in the model (i.e., variables associated with the outcome variable at *p* > .2) were removed from final models for simplicity. Detection-tolerance and the variance inflation factor (VIF) were used to assess multicollinearity before testing for mediation.

Given that parallel mediation models (i.e., a single model that includes 2 proposed mediators) require considerably larger sample sizes to achieve adequate power than single mediation models (Sim et al. 2022), the present study conducted 4 single mediation models to examine the indirect effect of baseline Faith on 15-month PCS and MCS via 3-month Meaning and Peace using the bias-corrected bootstrap test of indirect effects via the PROCESS-macro v 3.3 for SPSS (model 4) (Hayes 2013). To explore *changes* in Meaning and Peace as mediators of the association between Faith and health-related QoL, the 4 models were rerun including the baseline level of the mediator variable as a covariate. All mediation model results are presented as standardized regression coefficients.

Power was estimated using the method proposed by Pan et al. (2018) to determine the sample size needed to detect mediation effects using longitudinal data. Given moderate interclass correlations between measures at the 3 time points (ICC of .5), a sample size of 109 would allow a simple indirect effect to be detected as statistically significant at alpha of .05 with 80% power using a bootstrapped approach if the associations between the predictor and mediator and between the mediator and outcome were small-to-moderate (e.g., *β* = .26).

## Results

### Sample characteristics

Participant characteristics can be seen in Table 1. Most (85%) were still undergoing active treatment and 92% were within 6 weeks of their diagnosis at study entry. Most identified as Protestant Christian (*n* = 47, 41%), followed by Catholic (*n* = 31, 27%), and Other (*n* = 26, 23%), with few identifying as nonreligious (*n* = 6, 5%). In the parent study, there were 332 participants, but 222 had missing self-report data and were not included in these analyses. There were no systematic differences between those with complete self-report data (*N* = 110) and those without (*N* = 222) on self-report variables; yet there were 2 differences in demographic variables: those with incomplete data were younger (*M* = 48 vs. 51 years, *p* = .03) and less likely to have a college education (53% vs. 74% college educated, *p* < .001).

**Table 1.** Demographic and medical characteristics Table 1 long description.

Characteristics (*N* = 123)	M/N	SD/%
Age, M, SD	51.42	10.02
Ethnicity, *n*, %		
White/European American	75	64.0%
Hispanic/Latino	17	14.9
Black/African American	17	14.9
Asian American	5	4.4
Native American	1	0.9
Prefer not to answer	1	0.0
Marital status, *n*, %		
Married or living with partner	81	71.7
Divorced	13	11.4
Widowed	2	1.8
Never married	7	6.1
Prefer not to answer	5	4.4
Education, *n*, %		
Less than High School	4	3.5
High School Graduate	11	9.6
Some College	12	10.5
College Graduate	49	43.0
Graduate Degree	34	29.8
Prefer not to answer	4	3.5
Income, *n*, %		
≤ 75 K	46	40.3
> 75 K	54	47.4
Prefer not to answer	14	12.3
Employment, *n*, %		
Full-time	44	38.6
Part-time	22	19.3
Looking for work	14	13.2
Retired	30	26.3
Prefer not to answer	3	2.6
Religion, *n*, %		
Protestant	47	41.2
Catholic	31	27.2
Jewish	1	0.9
Other	26	22.8
Nonreligious	6	5.3
Prefer not to answer	3	2.6
Stage, *n*, %		
I	23	20.2
II	64	56.1
III	27	23.7
Time since diagnosis (d), *n*, %	23.36	31.02
Type of treatment planned, *n*, %		
Chemotherapy (currently on)	98	86.0
Radiation	10	8.8
Hormone therapy	4	3.5
Unknown	2	1.8
Menopausal, *n*, %	62	54.4

Means of and correlations among study variables at each time point are in Table 2. When examining bivariate correlations, baseline and 3-month Peace and Meaning were significantly positively associated with 15-month MCS and PCS (*r*’s > .3), whereas Faith was not. Additionally, when assessed at the same time point (baseline), MCS was strongly correlated with Peace (*r* = .61) and Meaning (*r* = .53), and Peace and Meaning were correlated with one another (*r* = .62), suggesting these measures are strongly correlated but likely represent distinct constructs.

**Table 2.** Means and correlations of study variables Table 2 long description.

				Baseline					3-Month			15-Month	
Time	Variable	Mean	SD	Faith	Meaning	Peace	MCS	PCS	Faith	Meaning	Peace	MCS	PCS
Baseline	Faith	13.62	3.33	1									
	Meaning	14.15	2.48	.340**	1								
	Peace	11.92	3.34	.425**	.622**	1							
	MCS^+^	48.65	8.69	.161	.529**	.612**	1						
	PCS^+^	44.91	10.29	−.046	.117	.244*	0.167	1					
3-Months	Faith	13.74	3.29	.857**	.357**	.452**	0.183	0.017	1				
	Meaning	13.96	2.30	.371**	.622**	.586**	.444**	0.186	.358**	1			
	Peace	12.39	3.27	.441**	.482**	.735**	.514**	.310**	.540**	.614**	1		
15-Months	MCS	50.58	9.51	.010	.221*	.389**	.519**	.405**	.096	.393**	.404**	1	
	PCS	48.17	10.00	−.019	.301**	.334**	.254**	.647**	.074	.380**	.342**	.426**	1

*Note:* ^+^*N* = 110, all other variables *N* = 114 (the participants included in the mediation analyses); *p < .05, **p < .01. PCS = Physical Component Summary; MCS = Mental Component Summary. Participants reported MCS and PCS similar to the normative mean of 50 (SD = 10) at study entry (MCS *M* = 48.7, SD = 8.7; PCS *M* = 44.9, SD = 10.3) and 15-months later (MCS *M* = 50.6, SD = 9.5; PCS *M* = 48.2, SD = 10.0). The average FACT-Sp total score at study entry (*M* = 39.7, SD = 7.3) and 3-months later (*M* = 40.1, SD = 7.3) was similar to the normative value reported by Peterman et al. (2002) (*M* = 38.5, SD = 8.1) and Canada et al. (2016) (*M* = 37.4, SD = 8.6). Similarly, the means of Faith, Meaning, and Peace subscales were comparable to previous studies of cancer survivors (Peterman et al. 2002; Canada et al. 2008, 2016).

### Determining covariates

#### Mental health-related QoL (Mental Component Summary; MCS)

Individuals employed at baseline reported higher 15-month MCS compared to unemployed individuals (*p* = .021; Cohen’s *d* = .49) and higher baseline income was associated with higher 15-month MCS (*r* = .37, *p* < .001). Additionally, baseline employment status, income, and MCS were associated with 15-month MCS at *p* < .2 in the initial mediation models, and so were retained in the final models in which MCS was the outcome variable; group was not associated with MCS (*p* > .6) in the initial mediation models and so was removed from the final models.

#### Physical health-related QoL (Physical Component Summary; PCS)

Younger age (*r* = −.22), less time since diagnosis (*r* = −.17), and higher income (*r* = .27) were associated with greater 15-month PCS (*p*’s < .05). Women who were employed at baseline (*p* = .001; Cohen’s *d* = .71), premenopausal (*p* = .040, Cohen’s *d* = .39), and college educated (*p* = .016, Cohen’s *d* = .63) reported higher 15-month PCS compared to women who were unemployed/retired, postmenopausal, without college degrees. Additionally, women who identified as being Catholic or Protestant reported higher 15-month PCS compared to those who indicated Other as their religious affiliation (*p* = .008). Income, education, menopausal status, religious affiliation, and baseline PCS were associated with 15-month PCS in initial mediation models at *p* < .2 and so were retained in final mediation models. Age, time since diagnosis, employment, and group were not associated with PCS (*p* > .6) in initial mediation models and so were removed from the final models.

### Indirect effects of baseline faith on 15-month health-related QoL (MCS and PCS) via 3-month meaning and peace and via change in 3-month meaning and peace

**Figure 1.**
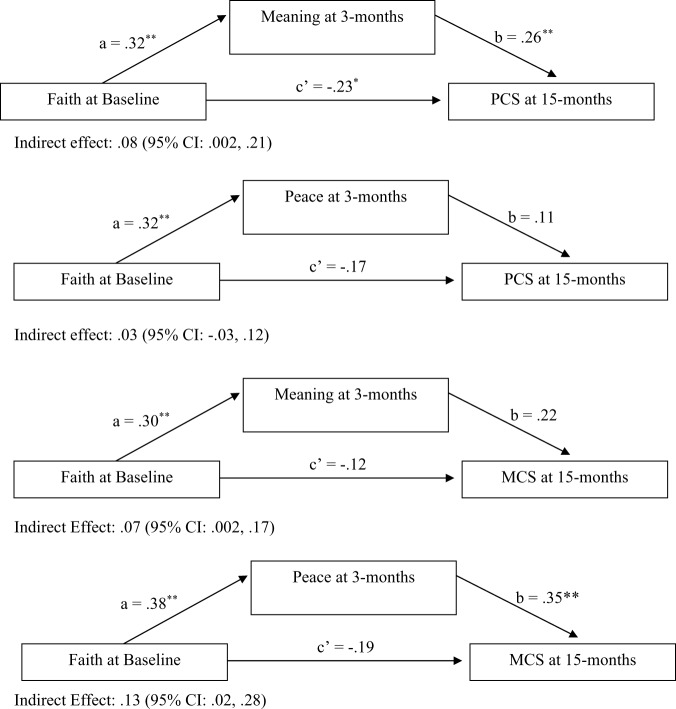
Mediation models exploring the indirect effect of baseline faith on 15-month health-related QOL through the proposed mediators of 3-month meaning and peace. Figure 1 long description.

*Note*: Values are standardized path coefficients. *p < .05 **p < .01; Covariates for models in which PCS is the outcome variable include income, education (college degree vs. no college degree), menopausal status, and religious affiliation (Catholic/Protestant, Other, and Nonreligious), and baseline PCS. Covariates for models in which MCS is the outcome variable include employment (vs. not employed), income, and baseline MCS.

Standardized regression coefficients for each mediation model can be seen in Figure 1. There was no direct effect of baseline Faith on 15-month MCS in the model that included 3-month Meaning as a mediator (*β* = −.12, *p* = .23); however, as hypothesized, the indirect effect of baseline Faith on 15-month MCS via 3-month Meaning was significant [*n* = 94, effect = .07 (95% CI: .002, .17)]. Specifically, baseline Faith was positively associated with 3-month Meaning (*β* = .31, *p* = .001), which was in turn positively associated with 15-month MCS (*β* = .22, *p* = .057). The model remained the same when controlling for baseline Meaning [*n* = 94, effect = .07 (95% CI: .001, .18)], with the association between 3-month Meaning and 15-month MCS becoming stronger (*β* = .36, *p* = .004), suggesting that *increases* in Meaning during active treatment were especially relevant for improving long-term mental health-related QoL.

There was no significant direct effect of baseline Faith on 15-month MCS in the model that included 3-month Peace as the mediator (*β* = −.19, *p* = .08); however, as hypothesized, the indirect effect of baseline Faith on 15-month MCS via 3-month Peace was significant [*n* = 93, effect = .13 (95% CI: .02, .28)]. Specifically, baseline Faith was positively associated with 3-month Peace (*β* = .38, *p* < .001), which was in turn positively associated with 15-month MCS (*β* = .35, *p* = .011). Contrary to hypotheses, these effects became nonsignificant when controlling for baseline Peace.

There was a direct effect of baseline Faith on 15-month PCS in the model that included 3-month Meaning as a moderator, such that higher baseline Faith was associated with lower 15-month PCS (*β* = −.23, *p* = .033). Additionally, as hypothesized, the indirect effect of baseline Faith on 15-month PCS via 3-month Meaning was significant [*n* = 92, effect = .08 (95% CI: .002, .21)]. Specifically, baseline Faith was positively associated with 3-month Meaning (*β* = .32, *p* = .010), which was in turn positively associated with 15-month PCS (*β* = .26, *p* = .006). The indirect effect became nonsignificant when controlling for baseline Meaning, but the negative direct effect of baseline Faith on 15-month PCS remained significant (*β* = −.22, *p* = .037)

There was no direct effect of baseline Faith on 15-month PCS in the model that included 3-month Peace as the mediator (*β* = −.17, *p* = .14), and contrary to hypothesis, the indirect effect of baseline Faith on 15-month PCS via 3-month Peace was also not significant [*n* = 91, .03 (95% CI: −.03, .12)]. Though, baseline Faith was positively associated with 3-month Peace (*β* = .32, *p* = .004), 3-month Peace was not significantly associated with 15-month PCS (*β* = .11, *p* = .32). These effects remained nonsignificant when controlling for baseline Peace.

## Discussion

This study examined how patient-reported spiritual well-being in newly diagnosed women with breast cancer undergoing chemotherapy was associated with health-related QoL over 1 year. As 90% of the sample was within the first 6 weeks of initial cancer diagnosis, these results contribute to the literature by clarifying the longitudinal impact of spirituality during the challenging early treatment period including during chemotherapy. Consistent with hypotheses and extending the findings of prior cross-sectional research, this study found that greater faith at baseline was associated with higher levels of meaning and peace 3 months later, which in turn was associated with greater *mental* health-related QoL assessed 15 months after study entry. Additionally, meaning, but not peace, mediated the association between baseline faith and *physical* health-related QoL, consistent with previous research suggesting a stronger association between meaning and physical QoL (Peterman et al. 2014) and other studies suggesting that meaning is a particularly robust mediator (Canada et al. 2023).

Thus, faith appears to be indirectly associated with subsequent health-related QoL via greater meaning and peace. Further, by controlling for baseline meaning in the model, we were able to demonstrate that the indirect association of faith on mental health-related QoL was also mediated by the *change* in meaning over time. In other words, greater faith was associated with an *increase* in meaning during active treatment, which led to greater mental health-related QoL 1 year later.

These findings replicate and extend prior work by several researchers who have found faith to be indirectly associated with health-related QoL via meaning and peace in cross-sectional samples (Edmondson et al. 2008; Park et al. 2008; Canada et al. 2016; Merluzzi et al. 2023). For example, Canada et al. (2016) highlighted the indirect role of faith in promoting well-being through existential factors such as meaning and peace in a large cross-sectional sample of cancer survivors. Using the same sample, Canada et al. (2023) reported that the positive associations of religious service attendance and certainty regarding one’s belief in God with mental and physical health QoL were mediated by meaning, further suggesting that religious faith informs one’s meaning-making in difficult circumstance, which in turn influence QoL. Like the current study, peace was not found to be a mediator in Canada et al.’s study (Canada et al. 2023).

Current results also align with Merluzzi et al. (2023), who found that surrendering control to a higher power – one specific aspect of faith – was indirectly associated with well-being through a combined meaning/peace variable in a cross-sectional sample of over 500 cancer survivors. The longitudinal nature of our study provides further evidence that one’s faith, or system of meaning, informs subsequent perceptions of meaning and peace, which in turn shape QoL.

Examining the direct association between baseline faith and 15-month QoL was not the primary goal of this study, given existing evidence that faith has a weaker direct association with QoL relative to meaning or peace (Canada et al. 2016) and the theoretical and empirical rational for examining faith in conjunction with meaning and peace when considering QoL outcomes (Park 2013). Thus, it is not surprising that 3 of the 4 mediation models did not support a direct association between Faith and QoL. However, one model did suggest a surprising direct negative association between Faith and subsequent physical health-related QoL: greater baseline faith was associated with *poorer* physical health-related QoL in the model including Meaning as the mediator. This suggests that, when variance in physical health-related QoL explained by Meaning is accounted for, the association between Faith and physical health-related QoL may be negative. In other words, faith in the absence of meaning may be unhelpful in terms of physical QoL. It is possible that individuals with high faith but low meaning might experience more spiritual distress that would impede health-promoting behaviors. However, this finding needs to be interpreted with caution due to the exploratory nature of these analyses.

### Limitations

Limitations of the current study are primarily related to selection of participants for the original Tibetan yoga study. The current sample was relatively financially wealthy, and greater income was associated with better mental and physical QoL. Older women and those with higher levels of education may be over-represented as they were more likely to provide complete self-report data in the parent study. This study also excluded women engaged in psychological treatment or support or who believed they needed psychological or psychiatric services. The current sample reported normative levels of QoL, particularly mental health-related QoL, which may also reflect selection bias for women who chose to join this study of a yoga intervention. It may have been more challenging to detect significant associations between aspects of spirituality and mental health-related QoL due to ceiling effects in this sample.

### Implications and conclusions

This study suggests that faith, understood as strength and comfort in one’s beliefs in the face of adversity, may be an important precursor to spiritual peace and especially meaning, which demonstrates robust associations with better long-term mental and physical QoL. Importantly, participants in this study were newly diagnosed and undergoing active treatment, suggesting that spiritual resources at the outset of the cancer experience can impact later functioning. One recent study suggests that meaning changed the most over time among cancer survivors (compared to faith and peace) and was positively associated with social support and adaptive coping, suggesting that this is a malleable factor (Park et al. 2024). Clinicians working with this population may benefit from attending to patients’ spiritual needs early in care, particularly to foster meaning and peace. Psychological interventions such as Meaning-Centered Psychotherapy (MCP) may be particularly useful in helping patients reconnect with a sense of meaning during cancer treatment. MCP is a brief, structured, cancer-specific psychological intervention designed to address loss of meaning, identity, and purpose after cancer diagnosis. In fact, in writing about how MCP might be useful for breast cancer survivors, Lichtenthal et al. (2017) echoed the current findings: “meaning is hypothesized to be both an intermediary outcome (a construct to be enhanced in its own right) and a mediator, driving improvement in multiple psychological distress outcomes (e.g., by redirecting attention toward meaningful activities)” (p. 57). MCP is flexible, allowing individuals to integrate their own religious or spiritual beliefs into the therapeutic process. Other interventions that may help to bolster meaning in cancer include Dignity Therapy and Acceptance and Commitment Therapy (Sauer et al. 2024; Seiler et al. 2024). Each of these interventions elicit the patient’s most important values and beliefs to encourage behaviors that are ultimately health-promoting.

The current study builds on a substantial body of research suggesting that religion and strength or comfort in one’s faith work through intermediary constructs of meaning and peace to improve cancer patients’ QoL. Longitudinal studies are especially needed to examine how religiosity, spirituality, and meaning evolve over time, how best to intervene upon these constructs, and what impact such changes have on individuals with cancer, their families, and the healthcare teams who support them.

## Table 1 Long description

The table summarizes demographic and medical characteristics for 123 participants, reporting mean and standard deviation for age and time since diagnosis, and counts with percentages for categorical variables. Mean age was 51.42 years with a standard deviation of 10.02. Ethnicity was mostly White or European American (75, 64.0%), with Hispanic or Latino and Black or African American each at 17 (14.9%), Asian American at 5 (4.4%), and Native American at 1 (0.9%). Most participants were married or living with a partner (81, 71.7%); smaller groups were divorced (13, 11.4%), never married (7, 6.1%), or widowed (2, 1.8%). Education was highest for college graduates (49, 43.0%) and graduate degrees (34, 29.8%), with fewer reporting high school or less. Income was split between 75 thousand or less (46, 40.3%) and above 75 thousand (54, 47.4%), with 14 (12.3%) preferring not to answer. Employment was most often full-time (44, 38.6%) or retired (30, 26.3%), and religion was most commonly Protestant (47, 41.2%) or Catholic (31, 27.2%). Disease stage was primarily stage two (64, 56.1%), followed by stage three (27, 23.7%) and stage one (23, 20.2%); time since diagnosis averaged 23.36 days with a standard deviation of 31.02. Most patients had chemotherapy currently underway (98, 86.0%), and 62 participants (54.4%) were menopausal; some categories include small “prefer not to answer” groups, so totals may not align perfectly across sections.

Navigate back to Table 1.

## Table 2 Long description

The table reports means and standard deviations for spirituality subscales (faith, meaning, peace) at baseline and 3 months, and health-related quality of life (mental and physical component scores) at baseline and 15 months, along with correlations within and across time points. At baseline, meaning and peace are strongly related to each other and both are moderately to strongly related to baseline mental health, while faith has weaker links to mental health. Baseline physical health shows little relationship with faith or meaning and only a small positive relationship with peace. Spirituality scores are stable from baseline to 3 months, with baseline faith strongly predicting 3-month faith and baseline peace strongly predicting 3-month peace. At 15 months, mental health is moderately related to baseline mental health and shows small to moderate positive relationships with baseline and 3-month meaning and peace, but little relationship with faith. At 15 months, physical health is strongly related to baseline physical health and shows moderate positive relationships with baseline and 3-month peace and with meaning, again with minimal relationship to faith. Correlations marked as statistically significant indicate more confidence in those associations, and sample size is slightly smaller for the baseline health measures than for other variables.

Navigate back to Table 2.

## Figure 1 Long description

Insufficient visual information to describe this element accurately.

Navigate back to Figure 1.

## References

[ref1] Ahmad N, Sinaii N, Panahi S, et al. (2022) The FACIT-Sp spiritual wellbeing scale: A factor analysis in patients with severe and/or life-limiting medical illnesses. *Annals of Palliative Medicine* 11(12), 3663673. doi:10.21037/apm-22-692. 36366899

[ref2] Bai M, Dixon J, Williams AL, et al. (2016) Exploring the individual patterns of spiritual well-being in people newly diagnosed with advanced cancer: A cluster analysis. *Quality of Life Research: An International Journal of Quality of Life Aspects of Treatment, Care, and Rehabiltation* 25(11), 2765–2773. doi:10.1007/s11136-016-1328-0. 27271809

[ref3] Bai M and Lazenby M (2015) A systematic review of associations between spiritual well-being and quality of life at the scale and factor levels in studies among patients with cancer. *Journal of Palliative Medicine* 18(3), 286–298. doi:10.1089/jpm.2014.0189. 25303461 PMC4348086

[ref4] Canada AL, Murphy PE, Fitchett G, et al. (2008) A 3‐factor model for the FACIT‐Sp. *Psycho-Oncology: Journal of the Psychological, Social and Behavioral Dimensions of Cancer* 17(9), 908–916. doi:10.1002/pon.1307. 18095260

[ref5] Canada AL, Murphy PE, Fitchett G, et al. (2016) Re-examining the contributions of faith, meaning, and peace to quality of life: A report from the American Cancer Society’s Studies of Cancer Survivors-II (SCS-II). *Annnals Behavioral Medicine* 50(1), 79–86. doi:10.1007/s12160-015-9735-y. 26384498

[ref6] Canada AL, Murphy PE, Stein K, et al. (2023) Assessing the impact of religious resources and struggle on well-being: A report from the American Cancer Society’s Study of Cancer Survivors-I. *Journal of Cancer Survivorship* 17(2), 360–369. doi:10.1007/s11764-022-01226-8. 35726114 PMC10084782

[ref7] Chaoul A, Milbury K, Spelman A et al. (2018) Randomized trial of Tibetan yoga in patients with breast cancer undergoing chemotherapy. *Cancer* 124(1), 36–45. doi:10.1002/cncr.30938. 28940301 PMC5735004

[ref8] Counted V, Possamai A and Meade T (2018) Relational spirituality and quality of life 2007 to 2017: An integrative research review. *Health and Quality of Life Outcomes* 16(1), 75. doi:10.1186/s12955-018-0895-x. 29690887 PMC5926536

[ref9] Davis LZ, Cuneo M, Thaker PH, et al. (2018) Changes in spiritual well‐being and psychological outcomes in ovarian cancer survivors. *Psycho‐Oncology* 27(2), 477–483. doi:10.1002/pon.4485. 28637083 PMC5740010

[ref10] Delgado-Guay MO, Palma A, Duarte E, et al. (2021) Association between spirituality, religiosity, spiritual pain, symptom distress, and quality of life among Latin American patients with advanced cancer: A multicenter study. *Journal of Palliative Medicine* 24(11), 1606–1615. doi:10.1089/jpm.2020.0776. 33844951 PMC9022128

[ref11] Edmondson D, Park CL, Blank TO, et al. (2008) Deconstructing spiritual well‐being: Existential well‐being and HRQOL in cancer survivors. *Psycho-Oncology: Journal of the Psychological, Social and Behavioral Dimensions of Cancer* 17(2), 161–169. doi:10.1002/pon.1197. 17506077

[ref12] Fairchild AJ and McDaniel HL (2017) Best (but oft-forgotten) practices: Mediation analysis1, 2. *The American Journal of Clinical Nutrition* 105(6), 1259–1271. doi:10.3945/ajcn.117.152546. 28446497 PMC5445681

[ref13] Hayes AF (2013) *Introduction to Mediation, Moderation, and Conditional Process Analysis: A Regression-based Approach*. New York, NY: Guilford Press.

[ref14] Hebert R, Zdaniuk B, Schulz R, et al. (2009) Positive and negative religious coping and well-being in women with breast cancer. *Journal of Palliative Medicine* 12(6), 537–545. doi:10.1089/jpm.2008.0250. 19508140 PMC2789454

[ref15] Heidary Z, Ghaemi M, Hossein Rashidi B, et al. (2023) Quality of life in breast cancer patients: A systematic review of the qualitative studies. *Cancer Control* 30, 10732748231168318. doi:10.1177/10732748231168318. 37082898 PMC10236425

[ref16] Koenig HG and Carey LB (2025) Approaches for analyzing the relationship between spirituality and health using measures contaminated with indicators of mental and social health. *Journal of Religion and Health* 64(2), 1276–1286. doi:10.1007/s10943-025-02249-y. 39808227

[ref17] Lichtenthal WG, Roberts KE, Jankauskaite G, et al. (2017) Meaning-centered group psychotherapy for breast cancer survivors. In Breitbart W (ed), *Meaning-Centered Psychotherapy in the Cancer Setting*. New York: Oxford University Press, 54–66.

[ref18] Maxwell SE and Cole DA (2007) Bias in cross-sectional analyses of longitudinal mediation. *Psychological Methods* 12(1), 23–44. doi:10.1037/1082-989X.12.1.23. 17402810

[ref19] Merluzzi TV, Salamanca-Balen N, Philip EJ, et al. (2023) “Letting go” - Relinquishing control of illness outcomes to God and quality of life: Meaning/peace as a mediating mechanism in religious coping with cancer. *Social Science & Medicine* 317, 115597. doi:10.1016/j.socscimed.2022.115597. 36535230 PMC9962851

[ref20] Montazeri A (2008) Health-related quality of life in breast cancer patients: A bibliographic review of the literature from 1974 to 2007. *Journal of Experimental & Clinical Cancer Research* 27, 1–31. doi:10.1186/1756-9966-27-32. PMC2543010 18759983

[ref21] Pan H, Liu S, Miao D, et al. (2018) Sample size determination for mediation analysis of longitudinal data. *BMC Medical Research Methodology* 18(1), 32. doi:10.1186/s12874-018-0473-2. 29580203 PMC5870539

[ref22] Park CL (2005) Religion as a meaning‐making framework in coping with life stress. *Journal of Social Issues* 61(4), 707–729. doi:10.1111/j.1540-4560.2005.00428.x.

[ref23] Park CL (2013) Religion and Meaning. In Paloutzian RF and Park CL (eds), *Handbook of the Psychology of Religion and Spirituality*, 2nd edn. New York, NY: Guilford Press, 357–379.

[ref24] Park CL, Magin ZE, Bellizzi KM, et al. (2024) Trajectories of cancer survivors’ spiritual well-being through the transition from treatment to early survivorship. *Psycho-Oncology* 33(12), e70040. doi:10.1002/pon.70040. 39632284 PMC11793928

[ref25] Park CL, Malone MR, Suresh D, et al. (2008) Coping, meaning in life, and quality of life in congestive heart failure patients. *Quality of Life Research* 17(1), 21–26. doi:10.1007/s11136-007-9279-0. 18034319

[ref26] Peres MFP, Kamei HH, Tobo PR, et al. (2018) Mechanisms behind religiosity and spirituality’s effect on mental health, quality of life and well-being. *Journal of Religion and Health* 57(5), 1842–1855. doi:10.1007/s10943-017-0400-6. 28444608

[ref27] Peteet JR and Balboni MJ (2013) Spirituality and religion in oncology. *Ca A Cancer Journal for Clinicians* 63(4), 280–289. doi:10.3322/caac.21187. 23625473

[ref28] Peterman AH, Fitchett G, Brady MJ, et al. (2002) Measuring spiritual well-being in people with cancer: the functional assessment of chronic illness therapy—Spiritual Well-being Scale (FACIT-Sp). *Annals of Behavioral Medicine* 24(1), 49–58. doi:10.1207/S15324796ABM2401_06. 12008794

[ref29] Peterman AH, Reeve CL, Winford EC, et al. (2014) Measuring meaning and peace with the FACIT–spiritual well-being scale: Distinction without a difference? *Psychological Assessment* 26(1), 127–137. doi:10.1037/a0034805. 24188147 PMC4081471

[ref30] Reulen RC, Zeegers MP, Jenkinson C, et al. (2006) The use of the SF-36 questionnaire in adult survivors of childhood cancer: Evaluation of data quality, score reliability, and scaling assumptions. *Health and Quality of Life Outcomes* 4(1), 77. doi:10.1186/1477-7525-4-77. 17022814 PMC1618832

[ref31] Sauer C, Haussmann A and Weissflog G (2024) The effects of acceptance and commitment therapy (ACT) on psychological and physical outcomes among cancer patients and survivors: An umbrella review. *Journal of Contextual Behavioral Science* 33, 100810. doi:10.1016/j.jcbs.2024.100810.

[ref32] Seiler A, Amann M, Hertler C, et al. (2024) Effects of dignity therapy on psychological distress and wellbeing of palliative care patients and family caregivers – a randomized controlled study. *BMC Palliative Care* 23(1), 73. doi:10.1186/s12904-024-01408-4. 38486192 PMC10938771

[ref33] Sim M, Kim S-Y and Suh Y (2022) Sample size requirements for simple and complex mediation models. *Educational and Psychological Measurement* 82(1), 76–106. doi:10.1177/00131644211003261. 34992307 PMC8725051

[ref34] Stefanek M, McDonald PG and Hess SA (2005) Religion, spirituality and cancer: current status and methodological challenges. *Psycho-Oncology: Journal of the Psychological, Social and Behavioral Dimensions of Cancer* 14(6), 450–463. doi:10.1002/pon.861. 15376283

[ref35] Walker SJ, Chen Y, Paik K, et al. (2017) The relationships between spiritual well-being, quality of life, and psychological factors before radiotherapy for prostate cancer. *Journal of Religion and Health* 56(5), 1846–1855. doi:10.1007/s10943-016-0352-2. 28039542

[ref36] Ware JEJ (1999) SF-36 health survey. In Maruish ME (ed), *The Use of Psychological Testing for Treatment Planning and Outcomes Assessment*. Hillsdale, NJ: Lawrence Erlbaum Associates, 1277–1246.

[ref37] Whitford HS and Olver IN (2012) The multidimensionality of spiritual wellbeing: Peace, meaning, and faith and their association with quality of life and coping in oncology. *Psycho-Oncology* 21(6), 602–610. doi:10.1002/pon.1937. 21370313

[ref38] Yanez B, Edmondson D, Stanton AL, et al. (2009) Facets of spirituality as predictors of adjustment to cancer: Relative contributions of having faith and finding meaning. *Journal of Consulting and Clinical Psychology* 77(4), 730–741. doi:10.1037/a0015820. 19634965 PMC2825181

